# State Plan Index: A Tool for Assessing the Quality of State Public Health Plans

**Published:** 2005-03-15

**Authors:** Frances D Butterfoss, Diane O Dunĕt

**Affiliations:** Health Promotion & Disease Prevention, Center for Pediatric Research, Eastern Virginia Medical School; Centers for Disease Control and Prevention, National Center for Chronic Disease Prevention and Health Promotion, Division of Nutrition and Physical Activity, Chronic Disease Nutrition Branch, Atlanta, Ga

## Abstract

**Introduction:**

The State Plan Index is an evaluation instrument that uses a Likert scale to assess 60 indicators of the quality of state public health plans. The State Plan Index was needed to enable evaluation of plans that were developed using a variety of public health planning models.

**Methods:**

Federal, state, and academic partners participated in developing and testing the instrument. The authors conducted a literature review, interviews with experts, and several rounds of formative evaluation to assess item inclusion, coverage, weighting, organization of items, and content validity. In two rounds of field testing, public health practitioners at the federal and state levels rated 10 state public health plans for obesity prevention.

**Results:**

Field-test raters took an average of two hours to rate a plan and indicated that the State Plan Index was "easy to use," "comprehensive," and "fair." Mean Cronbach a for components of the State Plan Index was 0.88 (median 0.93). Component scores among the 10 plans rated ranged from 0.2 to 4.8, indicating that raters made distinctions in quality among the components and the plans they rated. Correlations between component scores and overall scores were statistically significant (*P* < .001), except for one component.

**Conclusion:**

Public health professionals at the federal and state levels found the State Plan Index to be a useful tool for evaluating public health plans that were developed by states using various planning approaches. After the field tests, state staff reported adapting the State Plan Index for use as a planning tool, an evaluation tool for local plans, and a self-assessment tool for drafts of state plans. In addition, the State Plan Index can be revised easily for use in other chronic disease areas.

## Introduction

Many professionals encourage public health planning as a key step in addressing complex issues such as chronic disease (1). This is especially true when problems require long-term strategies and multiple approaches, such as changes in policy, the environment, or individual behavior. Yet despite the widely held assumption that planning is important and despite the investment of substantial resources in planning at state and community levels, a key question lingers: Do better plans lead to better health outcomes?

In the last 25 years, an array of public health community planning, health education, and program development models have been developed, including PRECEDE–PROCEED (PRECEDE = Predisposing, Reinforcing, and Enabling Constructs in Educational/Ecological Diagnosis and Evaluation, and PROCEED = Policy, Regulatory, and Organizational Constructs in Educational and Environmental Development) (2); MAPP (Mobilizing Action through Planning and Partnership) (3); PATCH (Planned Approach To Community Health) (4); CHIP (Community Health Improvement Program) (5); and the Six-Step Program Development Chain Model (6). Other public health planning models address particular public health strategies, such as the CDCynergy model for planning, managing, and evaluating public health communication programs (7) and Intervention Mapping for designing theory- and evidence-based health promotion programs (8). Still others are focused on planning for a particular public health problem, such as planning for Comprehensive Cancer Control (9) and Getting to Outcomes for substance abuse prevention (10).

The availability of different models provides public health practitioners with the flexibility not only to match the appropriate model with the intended goal but also to use a model that fits within the norms and expectations of an organization and that meets with acceptance in the community involved. A plan also may be designed using more than one model; Breckon et al assert that "model *elements* can be mixed or matched depending on what fits or is acceptable [italics added]" (11). The possibility of combining elements from different models offers greater flexibility in plan design but also creates a greater need for an evaluation instrument that remains reliable across a diverse and expanding body of public health plans.

Planning models generally prescribe a planning *process* rather than articulate desired attributes of a finished plan that is the outcome of such a process. To date, evaluation instruments have focused on assessment of planning processes (9,12,13) and methods to inventory or describe the content of community plans (14,15). Criteria to assess plan quality could be derived by implication from the concepts contained in each of the various planning models. However, this task is difficult and time-consuming for practitioners, who need to assess the quality of written plans regardless of the planning process(es) or model(s) used.

Although evaluation instruments for state plans are limited, tools to generally assess public health infrastructure or capacity have been developed and widely disseminated (16-18). For example, the School Health Index developed by the Centers for Disease Control and Prevention (CDC) (17) provides comprehensive questionnaires that schools can use as self-assessment and planning tools to improve the effectiveness of their health and safety policies and programs. On a larger scale, the National Public Health Performance Standards provide a framework for assessment of state and local public health systems (18).

The State Plan Index (SPI) was developed as part of the evaluation of the CDC's Nutrition and Physical Activity Program to Prevent Obesity and Other Chronic Diseases (Obesity Prevention Program) and is available from www.cdc.gov/nccdphp/dnpa/obesity/state_programs The CDC Obesity Prevention Program provides planning support and other assistance to states for obesity prevention and reduction. The SPI was needed to evaluate state plans that were developed by state public health practitioners and their community partners using a variety of public health planning models. In addition, to understand the relationship between plan quality and health outcomes in the long term, an evaluation instrument was needed to assess baseline plan quality. As described below, the SPI development process drew upon a wide array of existing public health planning models, tools, and resources.

## Methods

### Instrument development

Development of the SPI began in June 2002. The authors reviewed published professional public health literature on planning, community-based planning, plan assessment, and recommended planning methods, including but not limited to the references cited here. Key elements were gleaned from these public health planning models. In addition, planning processes that were considered critical across the models were identified. Other relevant published and unpublished materials were reviewed, including the CDC Obesity Prevention Program guidelines, reports, and existing state plans. One of the authors also conducted in-person key informant interviews with planning experts throughout the CDC's National Center for Chronic Disease Prevention and Health Promotion from the Divisions of Adolescent and School Health, Adult and Community Health, Cancer Prevention and Control, Diabetes Translation, Nutrition and Physical Activity, Oral Health, and Reproductive Health; the Office on Smoking and Health; and in the CDC's National Center for HIV, STD, and TB Prevention.

The authors then developed a set of key indicators of plan quality, intentionally incorporating the concept that a high-quality written plan should reflect both plan attributes as well as evidence of planning processes that experts had identified as critical. The list of key indicators was shared with state-level public health professionals who provided further suggestions for indicators and additional documents for review. State plans for comprehensive cancer control, cardiovascular health, and diabetes that were recommended as exemplary by practitioners were reviewed by the authors to identify common characteristics as potential SPI items. Through an iterative process, the State Plan Index evolved into a set of indicators grouped within major components.

In June 2003, telephone interviews were conducted by one of the authors with seven nationally recognized academic experts in strategic planning, public health, instrument development, and psychometrics. Federal and state public health practitioners and experts also participated in a formal review process to assess the SPI items proposed for inclusion, as well as in a formative evaluation process to recommend whether SPI items and components should be weighted equally. SPI items were also examined for coverage, overlap, weighting, and content validity. In total, approximately 100 public health representatives in federal, state, and academic settings provided suggestions for item inclusion and reviewed and commented on several preliminary drafts of the SPI. A list of the SPI components with the rationale for including each is presented in the Appendix.

### Sample, measures, testing, and refinement

A pilot-test version of the SPI, finalized in July 2003, consisted of 55 items grouped within nine components. A 5-point Likert scale was provided for each item, from 1 = low quality to 5 = high quality, with an additional "Not Addressed" option for each item. A similar Likert scale was provided to rate each component and the quality of the plan as a whole. "Not Addressed" was scored as 0 in the analyses described below. The authors conducted a pilot test of the instrument by independently rating two state plans. Based on this pilot test, wording of SPI items was clarified, and an assessment was made of the approximate time that would be needed to read and rate a plan.

The first of two field tests was conducted in July and August 2003 (Table 1). Nineteen raters participated in the first field test: 10 staff members from states funded through the CDC's Obesity Prevention Program, five staff members from other states who were members of the Association of State and Territorial Nutrition Directors, a paid independent public health consultant who rated all 10 plans, and three CDC staff members who rated five or 10 plans each. Raters were provided written instructions and a telephone orientation conducted by the authors to provide background information for the field test. No formal training was provided to raters, because the SPI was developed with the intention that it could be used by practitioners without the need for special training.

At the time of field testing, only 10 states had developed comprehensive plans for obesity prevention; nine of the 10 plans rated were from states funded through the CDC's Obesity Prevention Program. The plans were provided to the CDC or downloaded from the states' Web sites. As summarized in Table 1, each plan had four or five raters who provided a score for each item, each component, and the overall plan quality. Each plan was to have five raters, but two raters did not complete all ratings within the time allotted, resulting in a total of 46 rather than 50 ratings. Raters were assigned plans based on suggestions from the CDC Obesity Prevention Program staff members, who matched state plans with raters who were most likely to be unfamiliar with obesity prevention efforts in that state. Raters were requested to provide both numeric scores for each item as well as written feedback for each SPI component. In addition, written comments were solicited from the raters, and telephone debriefings were held with them to discuss any difficulties encountered in the rating process and to obtain suggestions for further refinements in the instrument.

Based upon the results of Field Test 1, minor changes in wording were made to the SPI, and five items were subdivided. To ensure that the changes to the SPI did not affect rating outcomes, Field Test 2 was conducted in November 2003 with a subset of the plans. Three plans were chosen to represent high-, low-, and average-scoring plans. The final 60-item version of the SPI was used by two raters — the same paid public health expert consultant from Field Test 1 and one new rater from the CDC Obesity Prevention Program who did not participate in Field Test 1.

### Analysis

Cronbach α was calculated for each component to assess whether items grouped within the component reliably measured the same dimension. Face validity for SPI items was determined by repeated review by federal, state, and academic planning and public health experts. Because no gold standard exists in the area of criterion validity (20), raters' overall plan scores were used as a proxy measure for criterion validity. Spearman rank correlation coefficients were calculated between raters' component scores and the overall score they assigned for each plan in Field Test 1. Although raters scored individual items before assigning an overall plan quality score, SPI instructions direct: "The [overall] score does not need to be an average of the [component] scores." Thus, raters were free to assign quality scores for each component and for the overall plan independently of their item-by-item ratings. To assess the consistency of plan ratings among raters while taking into account differences in plan quality, the interclass correlation coefficient (Shrout–Fleiss) was calculated for the overall plan score.

## Results

The final version of the SPI contains nine components: (A) Involvement of Stakeholders; (B) Presentation of Data on Disease Burden and Existing Efforts to Control Obesity; (C) Goals; (D) Objectives; (E) Selecting Population(s) and Strategies for Intervention; (F) Integration of Strategies with Other Programs and Implementation of Plan; (G) Resources for Implementation of Plan; (H) Evaluation; and (I) Accessibility of Plan. The Appendix provides a brief rationale for each component. A 5-point Likert scale ranging from 1 = low to 5 = high is used to score each item, each component, and the overall quality of a plan. A rating option of "Not Addressed" is also provided. Items are weighted equally, as are the nine SPI components.

The results of Field Test 1 showed a wide range of average score by component (0.2 to 4.8 on a 5.0 scale), indicating that raters made distinctions in quality among the components and among the plans rated. Raters took an average of 2.0 hours to review a plan and complete the SPI, compared to an average of 1.3 hours in the pilot test spent by the authors who had developed the SPI. The plans reviewed contained an average of 40 pages and generally included graphics and illustrative tables that noticeably reduced the volume of text. Thus, 2.0 hours was judged to be a reasonable length of time to review and rate a plan.

Overall, comments from field testers were very positive; raters commented that the SPI was "easy to use," "comprehensive," and "user-friendly" and that it "seemed fair" and made them "look at plans in a new and more systematic way." The most commonly reported problem was that raters were somewhat uncomfortable assigning a very low score when a plan had little detail. For example, several plans lacked detail regarding the development of financial or other resources for plan implementation. However, raters reported that states may have addressed resource issues even though detail was not provided in the plan reviewed.

Table 2 shows the coefficient of reliability (Cronbach α), calculated to assess whether items grouped within each component measured the same dimension. The average Cronbach α was 0.88, higher than the 0.8 level generally considered acceptable for social science data (21).

Table 2 also provides the Spearman rank correlation coefficient for each component, which indicates the correlation between component scores and overall plan scores that raters assigned in Field Test 1. All correlations were statistically significant at *P* < .001, except for Component G (Resources), a component that lacked detail in nearly all of the plans examined. Moderate to strong correlations were found between component scores and the overall plan quality score. The interclass correlation coefficient (Shrout–Fleiss) for Overall Plan Scores was 0.78 (skewed downward by low scores in the Resources component). The authors judged this to be an acceptable level of agreement among raters who rated the same plan. Data analyses were repeated for Field Test 2 with very similar results (data not shown).

During debriefing telephone conferences, raters were asked to comment further on their impressions of Component G (Resources) and their experience with the SPI ratings for plans that lacked detail. Some state staff reflected on their own plans, commenting that they had indeed addressed resources but were reluctant to reveal information about funding and resources outside of the planning group. They expressed concern that others might be inspired to tap into new resources and creative arrangements that planners had struggled to build. Despite these concerns, state and federal staff who participated in the debriefing agreed that the items in the SPI component for resources were appropriate and should be retained, especially if the SPI were to be translated from an evaluation tool into a guide for planning.

The authors also queried raters about whether they felt comfortable checking "Not Addressed" if an item was merely mentioned in a plan but inadequately addressed. Some raters noted their preference to provide written recommendations for improving a component or item, arguing that concrete suggestions were more important than "grades." However, other raters who checked some SPI boxes for low scores or "Not Addressed" noted that "grade inflation" could mask opportunities to strengthen a plan. To address this issue, future orientation sessions for SPI raters should stress the importance of using the SPI scoring system as a tool for providing clear feedback so that weak areas can be easily identified by states and appropriately addressed.

## Discussion

### Summary

The final SPI includes 60 items organized within nine components. The SPI can be used to evaluate plans developed using different public health planning models, thus providing a useful means of judging the quality of plans themselves. Moreover, although the SPI was developed for the CDC Obesity Prevention Program, most items can be easily adapted to other chronic disease areas. SPI pilot testers reported that the instrument was easy to use and consistent with the judgments they apply as public health professionals in assessing state plans. After the SPI field tests, some state staff, on their own initiative, used the SPI to self-assess their current plan and to guide development of action steps to address SPI items noted as weaknesses.

### Limitations

Although the SPI was judged as useful by experts in state, federal, and academic settings, several limitations remain. First, the concept of plan quality rests on the assumptions inherent in the public health models and literature reviewed. Second, because only 10 states had developed an obesity plan at the time of the SPI field testing, only these 10 plans were reviewed. Third, all testing was conducted on state obesity plans. Fourth, although the analyses generally showed high correlations between the component scores and the overall plan scores to corroborate criterion validity (except for Component G [Resources] that had missing data, as discussed above), the effect may be lessened because raters assigned their overall ratings after assessing individual items. Further, although the SPI is designed to help assess the quality of a written plan, even well-conceived plans may fail during implementation.

### Significance

Public health promotion models assume that quality planning will result in better health outcomes. Research in this area has been hampered by the lack of a useful instrument to measure plan quality at the state level. The proliferation of public health planning models and tools provides ideas to suit different planning groups and situations. If the widely held assumption that public health plans make a difference to health outcomes is correct, evaluation of the quality of the end product of planning (a written plan) is an important checkpoint. The SPI is grounded in theory, public health practice, and empirical field testing as well as in the expert opinions of state, federal, and academic collaborators.

Use of a systematic evaluation instrument also promotes the application of consistent standards in assessing state plans. Consistency has been embraced in the objective review panel process where written applications for federal funding are assessed against a detailed set of criteria. The SPI provides an evaluation tool that can be applied no matter who participated in the planning process or what planning approach was used.

Besides its use as an evaluation tool, the SPI has been adapted by state staff for use as a self-assessment tool. After participating in the CDC SPI field testing, one state staff member reported to the CDC that the state's obesity planning steering committee subsequently used the SPI to reassess its current written plan. Based on this review, the committee planned actions they would take to address potential weaknesses, such as adding faith-based organizations and consumers as stakeholders, restating plan objectives in measurable and time-based terms, and identifying ways to integrate obesity efforts with other chronic disease areas as well as across systems and agencies.

In an era of limited resources and increased accountability, linking public health efforts to health outcomes is more critical than ever. The SPI fills the need for an evaluation tool that can be used to systematically evaluate the quality of state plans. This assessment can ultimately be used to better understand the return on investment of resources devoted to planning.

Perhaps most importantly, the SPI provides straightforward, succinct guidance to public health practitioners embarking on a new planning process. Many of the practitioners who participated in the pilot test remarked that the SPI would have been very helpful to them if it had been available when their obesity program planning efforts were launched. As public health practitioners continue to engage in planning to address the growing burden of chronic disease in the United States, we hope that the SPI will prove a useful tool to guide and evaluate planning.

## Figures and Tables

**Table 1:** Summary of State Plan Index (SPI) Pilot Test and Field Tests, 2003

**Pilot Test** 55-item prototype version of SPI	Two authors each rate two state plans to assess usability of SPI format, clarity of wording, and time needed to read and rate a state plan. These two ratings were not included in statistical analyses reported here.
**Field Test 1** 55-item field test version of SPI	Ten state obesity plans, each rated by four to five raters from a pool of 19 raters. Each plan was to be rated by: one member of the Association of State and Territorial Public Health Nutrition Directors (from states *not* receiving CDC funding for obesity). one volunteer peer rater from a state receiving CDC funding for obesity one paid public health expert consultant who rated all 10 plans one CDC staff member from the Obesity Prevention Program who rated all 10 plans one of two other CDC staff members on the Obesity Prevention Program team who each rated five plans Number of plans rated = 46. Four states had four rather than five ratings because some ratings were not completed in the allotted time.
**Field Test 2** 60-item final version of SPI	Three state plans (chosen to represent high-, low-, and average-scoring plans from Field Test 1) were rated by the same paid public health expert consultant from Field Test 1 and one new rater from the CDC Obesity Prevention Program team who did not participate in Field Test 1. Number of plans rated for analysis = 6.

**Table 2:** Results of Field Test 1 of State Plan Index^a^, 2003

**State Plan Index Component**		**Reliability of Items Within Each Component**	**Correlation Between Component Score and Overall Plan Score**
		**Cronbach α**	**Spearman rank correlation coefficient (*P*) **
A	Stakeholders	0.93	0.49 (<.001)
B	Data on Disease Burden	0.92	0.62 (<.001)
C	Goals	0.99	0.70 (<.001)
D	Objectives	0.95	0.70 (<.001)
E	Strategies for Intervention	0.70	0.57 (<.001)
F	Integration of Strategies	0.87	0.52 (<.001)
G	Resources	0.68	0.07 (.65)
H	Evaluation	0.94	0.54 (<.001)
I	Accessibility	0.95	0.62 (<.001)
Overall Plan Score		0.83	Does not apply
Mean across components		0.88	0.54
Median of component scores		0.93	0.57

a Field Test 1 included 10 state plans, 46 ratings, and 19 raters.

## Appendix

- **Involvement of Stakeholders.** Early involvement increases the likelihood that stakeholders will develop a sense of ownership in the plan and a commitment to making it succeed. The different experiences and perspectives that partners bring will help ensure that the plan is responsive to the needs of all segments of the population. Each partner brings its own contacts and constituents, widening the base of support for the plan and increasing its credibility across the state. Community planning models emphasize the need for meaningful involvement of stakeholders, with some models designed for community-led planning. (See for example, MAPP [3].)
- **Presentation of Data on Disease Burden and Existing Efforts to Control Obesity.** Evidence-based public health practice must include a systematic examination of data on disease burden for population subgroups. Assessing existing resources that address a public health problem identifies opportunities for partnership and the potential to leverage additional resources. The use of reliable data sources lends credibility to the planning process. Evidence-based planning models emphasize the need for data to inform decision making. (See for example, PRECEED–PROCEED [2].)
- **Goals.** Goals provide a vision of what planners intend to achieve. Because planning itself consumes time and other resources, something important should be gained. Goals should unambiguously convey that something new is intended that is likely to lead to desired change in health status indicators. Tools based on community planning models have been developed to assist in developing goals, such as The Community Tool Box (19).
- **Objectives.** Objectives should be specific, measurable, achievable, results-oriented, time-phased, and logically organized. They should be consistent with the overall public health priorities of the state and tied directly to the goals specified in the plan. As with goals, tools that support planning models provide guidance on developing and writing sound objectives (19).
- **Selecting Population(s) and Strategies for Intervention.** Advances in social marketing applied to public health have contributed to the design of interventions better matched to the intended audience. Many planning models emphasize the importance of understanding a community and the unique attributes of its members before selecting strategies. (See, for example, CDCynergy [7].) Setting criteria for a systematic selection of interventions to be undertaken supports an evidence-based approach to public health. Although disease burden may figure prominently among the criteria used to select interventions, other criteria may be even more important, for example, political factors in a community or a subgroup’s readiness to change. Documenting the rationale for selecting strategies clarifies the planning group’s decision making process and informs plan implementers who become involved later.
- **Integration of Strategies with Other Programs and Implementation of Plan.** Public health partnerships and collaborations are key strategies to leverage limited resources. Often, however, a disadvantage with partnerships is having less direct control of action steps. Planning for systematic assessment of implementation steps helps ensure that a plan is carried out as designed and provides feedback useful for midcourse correction. Planning models may emphasize the need to consider how new strategies can be integrated into existing infrastructure. (See, for example, CHIP [5].)
- **Resources for Implementation of Plan.** A plan may serve little purpose unless planners address how to locate, maintain, and sustain resources needed to implement the plan. Although this step is not often explicitly addressed in planning models, public health practitioners provided many examples of promising new initiatives that terminated because of the lack of resources that could sustain efforts for a time period long enough to achieve intended outcomes. In an era when public health resources are stretched thin, planners must consider what resources are currently available as well as what would be needed to implement the plan.
- **Evaluation.** Virtually every planning model reviewed for this study identified evaluation as an important and useful activity. Some planning models also emphasize the importance of incorporating evaluation into a planning process. (See, for example, “Getting to Outcomes” [10].) As part of planning, measures of success can be identified and systems set in place to monitor progress and identify problems once plan implementation begins. Because planning groups may disband after a plan is written, planners should identify those who will carry out an evaluation and the audience for evaluation information.
- **Accessibility of Plan.** Just as varied planning models may be used, a written plan may have several different audiences. A good plan should be understandable and useful. As much as possible, the plan should be designed to elicit interest and support in the reader. Arrangements for distribution of a plan should be made early to ensure timely dissemination to those who can contribute to the plan’s implementation and success.
